# Automated Imaging and Analysis of Platelet, Coagulation and Fibrinolysis Activities Using a Novel Flow Chip-Based System at Physiological Temperature

**DOI:** 10.3390/mi16111253

**Published:** 2025-10-31

**Authors:** Xiang Gui, Bibian M. E. Tullemans, Bas de Laat, Johan W. M. Heemskerk, Frauke Swieringa

**Affiliations:** 1 Synapse Research Institute, Kon. Emmaplein 9, 6217 KD Maastricht, The Netherlands; g.xiang@thrombin.org (X.G.); b.tullemans@thrombin.org (B.M.E.T.); b.delaat@thrombin.org (B.d.L.); j.heemskerk@thrombin.org (J.W.M.H.); 2 Department of Biochemistry, Cardiovascular Research Institute Maastricht (CARIM), Maastricht University, 6229 ER Maastricht, The Netherlands

**Keywords:** coagulation, fibrinolysis, flow chip, microfluidic, platelet

## Abstract

Conventional whole-blood flow assays for quantifying thrombus formation are typically performed at room temperature and are technically demanding, which limits their translational applicability. We engineered a novel, disposable, mountable, and single-channel microfluidic chip (MC-2S), which is based on the Maastricht chamber (MC) and designed for automated evaluation of platelet function, coagulation and fibrinolysis under physiological conditions. The MC-2S chip allows customizable choices of thrombogenic surfaces, such as collagen and tissue factor. The chip was used in combination with an adapted, 1.3 kg brightfield/fluorescence microscope, operating at physiological temperature (37 °C), and with scripts for automated multicolor analysis of image features. The integrated system enables a robust, rapid, and high-content quantification of the kinetics of thrombus formation and dissolution. In platelet-sensitive mode, MC-2S demonstrated high sensitivity to antiplatelet therapy with aspirin or cangrelor. In coagulation-sensitive mode, it detected the anticoagulant effect of rivaroxaban plus its reversal by andexanet-α. In fibrinolysis-sensitive mode, it monitored tissue-type plasminogen activator-induced thrombus dissolution, inhibited by tranexamic acid. Collectively, the MC-2S platform was found to provide a versatile, physiologically relevant tool for functional hemostasis testing, with high potential for the acute and subacute evaluation of patient blood samples in the context of bleeding disorders, thrombosis risk, and drug monitoring.

## 1. Introduction

The hemostatic process occurs in the dynamic environment of flowing blood, where shear forces critically regulate platelet adhesion, activation and aggregation, along with local accumulation of (anti)coagulant factors. To mimic physiology, it is relevant to assay these processes under flow and body temperature, and to allow interactions between platelets, coagulation and fibrinolysis, which can only partly be captured in static tests.

The thrombus-forming process determined in flow-based perfusion measurements of blood from genetically modified mice using collagen surfaces has appeared to reflect arterial thrombus formation and tail bleeding outcomes in vivo [1,2]. Under non-coagulant conditions, such microfluidic measurements were able to integrate quantitative and qualitative platelet traits, producing contracting platelet aggregates [3]. For human blood, shear-dependent platelet activation has been assessed with other devices, such as the platelet-function analyzer (PFA)-200 and the total thrombus-formation analysis system (T-TAS), both of diagnostic value [4,5]. However, their sensitivity appeared to be restricted to severe primary hemostasis defects, with limited identification of for instance mild forms of von Willebrand disease or mild platelet function disorders [6,7]. To overcome these limitations, several microfluidic flow devices have been developed for both research and point-of-care blood testing [8,9,10,11,12]. Among these, the parallel-plate Maastricht chamber-1 (MC-1) was found to be effective in detecting congenital and acquired platelet function impairments in blood samples from knockout mice [1,13,14], or from human subjects, e.g., revealing the effects of gene sequence variance in super-enhancers affecting platelet function [15,16]. In addition, MC-1 was applied in the evaluation of antithrombotic potential of certain clinically used drugs [17,18]. However, limitations of the MC-1 set up were its dependency on a bulky microscopy system and the operation at room temperature (RT), which reduced sensitivity to the widely used antiplatelet drug aspirin [19].

Recent emerging microfluidic approaches enabled the simultaneous assessment of platelet adhesion, fibrin formation and fibrinolysis under flow. By combining immobilized collagen and tissue factor as substrates, hemostatic insufficiencies could be detected in patients with coagulation factor disorders [20], while fibrinolytic activities were quantified in the presence of fibrinolysis trigger [21]. Building on these developments, we upgraded the MC-1 device by using a new disposable flow chip, combined with a compact microscope and operating at physiological temperature. In addition, we developed a script for automated image analysis, thereby facilitating a user-friendly, point-of-care testing of major hemostatic processes.

## 2. Materials and Methods

### 2.1. Materials

Glass coverslips (24 × 60 mm) for microscopy were obtained from Thermo-Fisher (Breda, The Netherlands). Type I collagen (Horm-type) was purchased from Takeda (Hoofddorp, The Netherlands); tissue factor (Innovin) from Siemens Healthineers (Erlangen, Germany); bovine serum albumin (BSA) and aspirin from Sigma-Aldrich; 3′,3″-dihexyl oxacarbocyanine iodide (DiOC_6_) from Anaspec (Reeuwijk, The Netherlands); Alexa Fluor 546 (AF546)-labeled fibrinogen from Invitrogen-Thermo-Fisher (Eugene, OR, USA); cangrelor from Chiesi Pharmaceuticals (Amsterdam, The Netherlands); rivaroxaban from Bayer (Leverkusen, Germany); and andexanet-α from Santa Cruz Biotechnology (Heidelberg, Germany). Tissue-type plasminogen activator (t-PA) came from Boehringer Ingelheim (Ingelheim am Rhein, Germany) and tranexamic acid from Sigma-Aldrich (St. Louis, MO, USA). Other materials were obtained from sources described elsewhere [22].

### 2.2. Blood Donors and Blood Collection

Blood was obtained from healthy control subjects after full informed consent in accordance with the Declaration of Helsinki, and the Netherlands Code of Conduct for Medical Research and Local Procedures. Studies were approved by the local Medical Ethics Committee (NL52640.068.15/METC152015). Donors did not take medication affecting platelet function or coagulation at least two weeks prior to blood donation.

### 2.3. Design of Microfluidic MC-2S Device

For integrated measurements of whole-blood thrombus formation and dissolution at physiological temperature (37 °C), we developed a disposable flow chip, labeled as MC-2S (Synapse Research Institute, Maastricht). Flow channel dimensions in the chip were 30 mm length, 2 mm width and 50 µm height. The high width/height ratio used limited disturbing edge effects [23,24]. MC-2S retained several key features of the MC-1 parallel-plate chamber [20], including user-defined microspot coating with thrombogenic substances and a dual shallow inlet channel to allow full mixing of blood with recalcification buffer under coagulant conditions. This is further shown in Figure S1. In particular, the inlet and outlet ports had a 15° angle with the channel, whilst converting the flow profile from tubular to planar, to facilitate blood mixing and reduce flow disturbances [20].

**Figure 1:**
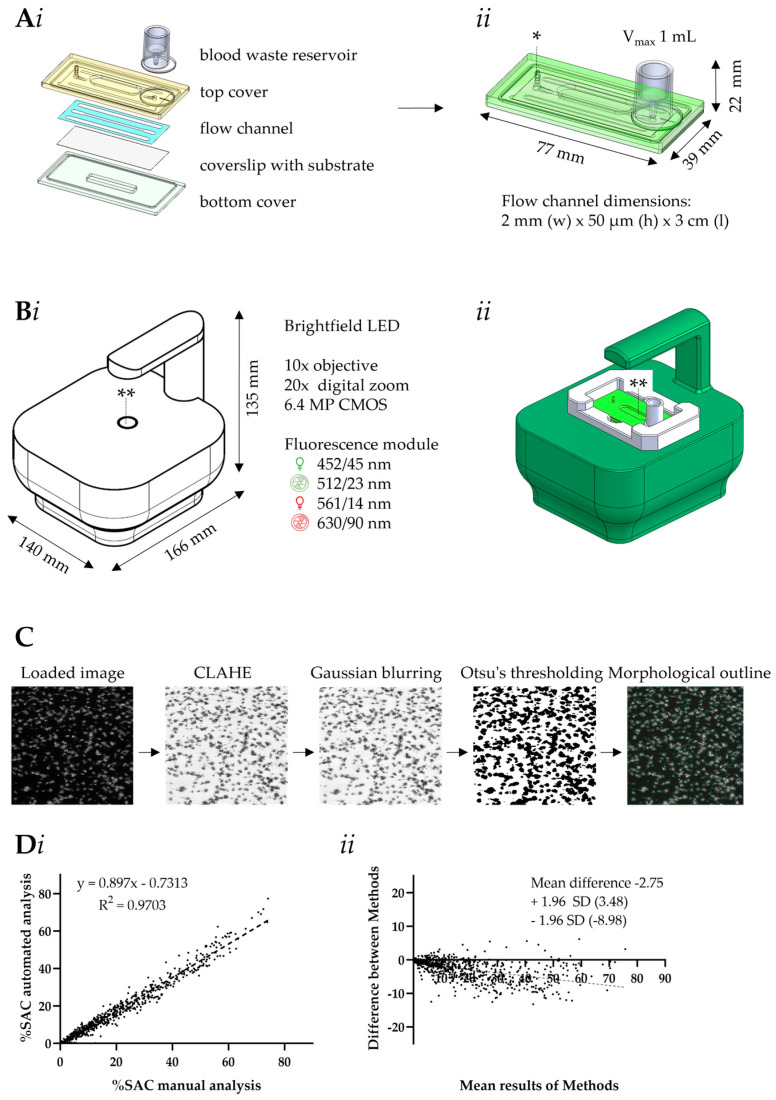
Drawing of MC-2S microchip, modified Cytosmart Lux3 microscope, and new script for image analysis. (**A**) Separate layers of the MC-2S chip (**i**) and the assembled chip (**ii**). The top layer contained a luer-lock connector to 1 mL syringe and a blood waste collector; a high-precision adhesive liner with microchannel notch (2 mm width × 50 μm height × 3 cm length); a glass coverslip spotted with thrombogenic material; and a bottom protection cover. The sign * shows the connection of chip to pump via G19 needle syringe (1 mm diameter). (**Bi**) Cytosmart microscope equipped with 10× objective, 20× digital zoom and 6.4 MP CMOS monochrome camera. The microscope allowed brightfield and fluorescence imaging with green and red fluorescence filter cubes. The sign ** shows the detection area (**Bii**) MC-2S chip placed on microscope. (**C**) Stepwise results of processed images by developed script. (**Di**) Regression analysis of quantified %SAC, as determined manually and by the script. (**Dii**) Bland-Altman plot of %SAC assessed manually or automatically. The *x*-axis shows the mean of the two methods and the *y*-axis shows the difference between methods. Indicated is a mean difference (bias) of −2.75, and the 95% limits of agreement were from −8.98 to +3.48. The data were from 48 measurements from eight donors, encompassing 692 pairwise analyzed time-lapse images.

Major innovations included: (i) an integrated blood reservoir; (ii) a slim, low-volume design for reduced blood input; and (iii) a rapid chip assembly using an adhesive strip layer (Figure 1A). The system further operated with a portable brightfield and fluorescence microscope, thermostated at 37 °C, in combination with a dedicated script for automated, multicolor, and brightfield image analysis (Figure 1B–D).

The blood flow for operation at certain wall-shear rate was calculated from the equation [23]: γ = (6 × Q)/(b × d^2^), where γ = shear rate (s^−1^); Q = volume flow rate (cm^3^/s, =pump rate); b = width (cm); and d = depth (cm). Hence, the 2-mm wide MC-2S chamber required 500 µL blood for a 10 min perfusion at 1000 s^−1^, which is less than the 750 µL required with the MC-1 chamber.

For automated brightfield and fluorescence microscopy, a Cytosmart Lux3 microscope (166 × 140 × 135 mm, 1.3 kg, Axion Biosystems, Atlanta, GA, USA) was adapted by Synapse Research Institute (Figure 1B). The microscope contained a 10× objective plus 20× digital zoom and incorporated a 6.4 MP CMOS monochrome camera (0.15 numerical aperture). Other optical characteristics were a 7 mm *z*-axis focus range and 7–10 µm depth of field. The Cytosmart microscope allowed brightfield image detection, and contained green (excitation 452/45, emission 512/23 nm) and red (excitation 561/14, emission 630/90 nm) fluorescence filter cubes. An adapted temperature-controlled housing maintained the chip at temperature of 37 °C. Temperature stability was achieved by a heating element connected to a temperature control module; this was calibrated to achieve 37 ± 1 °C prior to and during blood flow [19]. Temperature was monitored using a calibrated thermal imaging camera (sensitivity < 1 °C). The illumination exposure was set at 20 ms for brightfield, at 500 ms for green light (gain 30, intensity 80%) and at 500 ms for red light (gain 30, intensity 80%). Once initiated, the system performs time-lapse brightfield and fluorescence imaging at predefined intervals. Images were collected at 8-bits and 1040 × 1040 pixels, representing 728 × 728 µm.

Flow perfusion was performed by syringe-based injection of blood using one or two pulse-free nanopumps (Model 11 Plus, 70–2212, Harvard Apparatus, Holliston, MA, USA) in push mode. Of note, the chip design also allows use in a pull mode, where blood is added to the reservoir and drawn through the chip using the syringe, based on the channels symmetrical geometry, ensuring same flow patterns in either direction.

For coagulation experiments, citrated blood was continuously mixed with recalcification medium to achieve a homogeneous parallel-plate flow profile in the chamber, such as detailed in Figure S1.

### 2.4. Scripts for Automated Microscopic Image Analysis

The recorded series of gray images were automatically analyzed for platelet deposition, using a script written in Python 3.9.10 (Figure 1C), and accessible in OpenCV (Open Computer Vision Library). After loading a time series of raw 8-bit images (brightfield or single color fluorescence), the images were analyzed using the following steps: (i) image preprocessing by contrast enhancement (CLAHE) for edge detection, (ii) Gaussian blurring to reduce noise and smoothen local intensities variations, (iii) image segmentation by Otsu thresholding from a binary mask, and (iv) feature detection and quantification. The script allowed a quality check by providing morphological outlines of the identified features. Automated outcomes were checked with the original early, mid and end-point images. Quantitative outcome metrics were a list of feature sizes, and a cumulative curve of integrated features as percentage surface area coverage (%SAC). The script code is available on request.

The comparative script for manual image analysis [22] was operated by standardized journals, using the ImageJ software (version 1.53c: Rasband, NIH, Bethesda, MD, USA). Auto-enhanced images were filtered vertically and horizontally, and the thresholds were set manually. The resulting binary images were subjected to a close-and-open filter (morphology dilatation to probe and expand the relevant features), which resulted in identified regions of single or clustered adhered platelets. Image analysis included count and mark of features, an overlay of processed image on the original image, and the option to repeat steps and reapply manual threshold setting by user, if necessary. The comparison of automated and manual analysis indicated a high R^2^ of 0.97 (Figure 1Di), while Bland-Altman analysis showed a reasonable bias (Figure 1Dii).

The script outcomes produced quantitative information on platelet deposition (P1) and formed fibrin (P2) as percentage surface area coverage (%SAC). Based on earlier image analyses [23], auto-enhanced brightfield images were furthermore scored (observers blinded) for a thrombus morphological score 0–5 (P3, ranging from single platelet adhesion to full thrombus formation), and a thrombus contraction score 0–3 (P4, reflecting the contraction of platelet aggregates).

### 2.5. Quantification by MC-2S of Platelet, Coagulation and Fibrinolysis Activities

For use with MC-2S chips, degreased glass coverslips were coated with a single collagen type I (Horm type; 50 μg/mL, 10 μL) spot of 4.5 mm in diameter to ensure covering of the complete microfluidic chamber (and field of view). For non-coagulation experiments, coated coverslips were washed with water and dried under nitrogen. For coagulation and fibrinolysis experiments, the dried collagen-coated coverslips were mounted onto a flow channel, and post-coated with tissue factor (500 pM). Once assembled, the MC-2S chips were prerinsed for 30 min with 1% BSA in Hepes buffer pH 7.45 (136 mM NaCl, 2.7 mM KCl, 10 mM Hepes, 2 mM MgCl_2_). For studies with MC-1 chambers, degreased glass coverslips were similarly coated with collagen type I, followed by tissue factor as indicated.

Blood samples were preincubated with indicated drugs for 5 min at 37 °C, such as detailed in Table S1. After preincubation, the blood was labeled with 1 μg/mL DiOC_6_ (platelet stain) and 15 μg/mL AF546-fibrinogen (fibrin stain), both in a 1:100 volume ratio.

In the non-coagulation mode, blood was perfused (routinely at 1000 s^−1^) without recalcification for 8 min to achieve optimal platelet deposition and assess all platelet functional responses (single platelet adhesion—aggregate formation—active contracted thrombus formation).

In the coagulation and fibrinolysis mode, the blood sample was continuously mixed with recalcification medium (Hepes buffer pH 7.45, 63.2 mM CaCl_2_, 31.5 mM MgCl_2_, 9:1 volume ratio) for 10 min, which allowed massive fluorescent fibrin formation (>50% SAC). When fibrinolysis was measured, t-PA was present and blood perfusion was after 6 min continued for another 8 min with Hepes buffer, to allow major fibrin label decrease. Assay sensitivity of the processes was checked using clinically relevant therapeutics (Table S1). Image capturing in multicolor was continuously during the flow runs.

### 2.6. Data Analysis

Graphpad Prism version 8 (La Jolla, CA, USA) was used for analysis of the data. Data were checked for outliers. Statistical tests (non-parametric) were indicated and the differences with *p*-values <0.05 were considered to be significant.

## 3. Results

### 3.1. Platelet Activation to Thrombus Formation Assessed with MC-2S

To determine collagen-induced thrombus formation under shear conditions (arterial wall-shear rate of 1000 s^−1^) at controlled physiological temperature of 37 °C, we employed newly designed MC-2S disposable flow chips (Figure 1A). The chips were mounted onto a custom remodeled Cytosmart Lux3 microscope and connected to a computer enabling automated acquisition of time series of brightfield and dual-color fluorescence images (Figure 1B). Performance of the automated image analysis script (Figure 1C), developed in Python 3.9.10, was validated by comparison with a manual image analysis. Linear regression of the datasets yielded a high R^2^ of 0.97 (Figure 1D). While it is common practice to perform microfluidic blood perfusion experiments at RT, the present system also allowed experimentation at physiological temperature, thereby increasing the physiological relevance.

**Figure 2:**
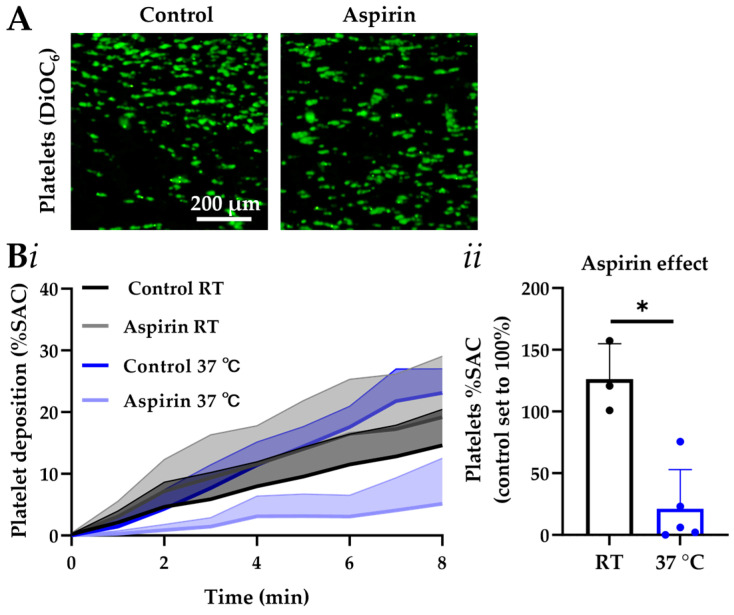

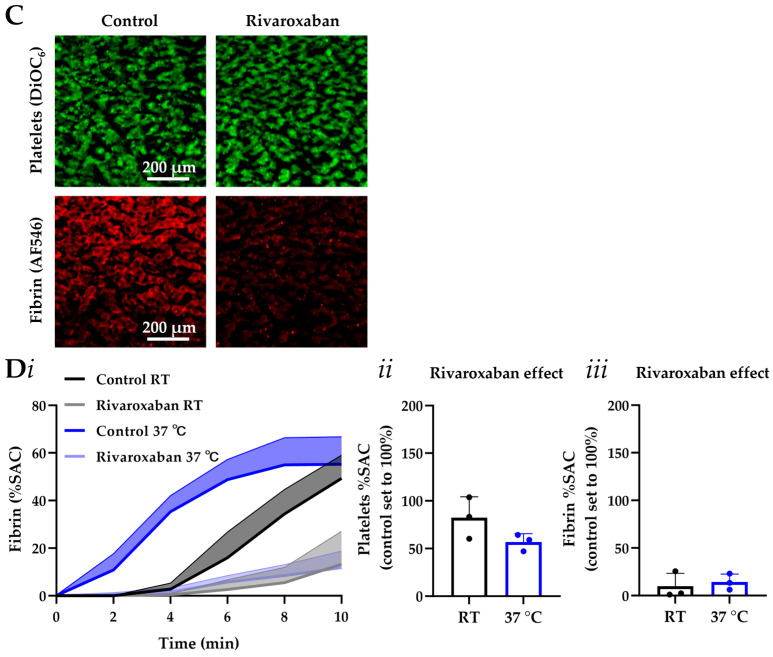
The MC-2S assessment of platelet thrombus and fibrin formation under flow and (non)coagulant conditions at room or physiological temperature. (**A**,**B**) Citrated blood samples were pretreated with vehicle control medium or aspirin (100 μM) for 5 min. After labeling with DiOC_6_ (1 μg/mL), the blood was flowed through MC-2S chips coated with collagen-I for 8 min at wall-shear rate of 1000 s^−1^ (37 °C or room temperature, RT). Recalcification was not applied. Image capture was every 5 s. (**A**) Representative images of platelet thrombi formed after 8 min at RT (bar = 200 μm). (**Bi**) Quantified traces of platelet deposition. Lower bold line indicates mean %SAC; upper line represents SD value. (**Bii**) Quantified effect of aspirin on platelet deposition after 8 min. (**C**,**D**) Citrated blood samples were preincubated for 5 min with rivaroxaban (600 nM). MC-2S chips were coated with collagen-I and tissue factor. After labeling with DiOC_6_ (1 μg/mL) and AF546-fibrinogen (15 μg/mL), blood was flowed under continuous recalcification for 10 min at 1000 s^−1^ (37 °C or RT). Two-color fluorescence images were captured every 10 s. (**C**) Representative images of platelet and fibrin thrombi formed after 10 min at RT (bar = 200 μm). (**D**) Time traces of fibrin formation (**i**). The lower bold line indicates mean %SAC and the upper line represents corresponding SD value. Effect of rivaroxaban on platelet deposition (**ii**) and fibrin formation (**iii**) at selected endpoint. The Mann-Whitney test with means + SD (*n* ≥ 3) was used, and the significant values were indicated by * *p*-value < 0.05.

Using this set up, we demonstrated that the higher temperature of 37 °C critically modulated platelet treatment sensitivity to the commonly used drug, aspirin (Figure 2). Aspirin inhibits platelet cyclooxygenase and thromboxane synthase, thereby blocking autocrine thromboxane A_2_ production [19]. Platelet responsiveness to aspirin was markedly enhanced at 37 °C compared to RT (Figure 2A,B). At 37 °C aspirin reduced platelet deposition by 78% from 23.1%SAC (surface area coverage) to 5.2%SAC (*p*-value < 0.05), while at RT no significant effect was seen. Platelet-dependent fibrin formation relying on coagulation was tested using the factor X inhibitor, rivaroxaban, as a widely used direct oral anticoagulant (DOAC). The fibrin-reducing effect of factor Xa inhibition was accelerated but not otherwise altered by the higher temperature (Figure 2C,D).

### 3.2. MC-2S Chip for Platelet Function Testing

**Figure 3:**
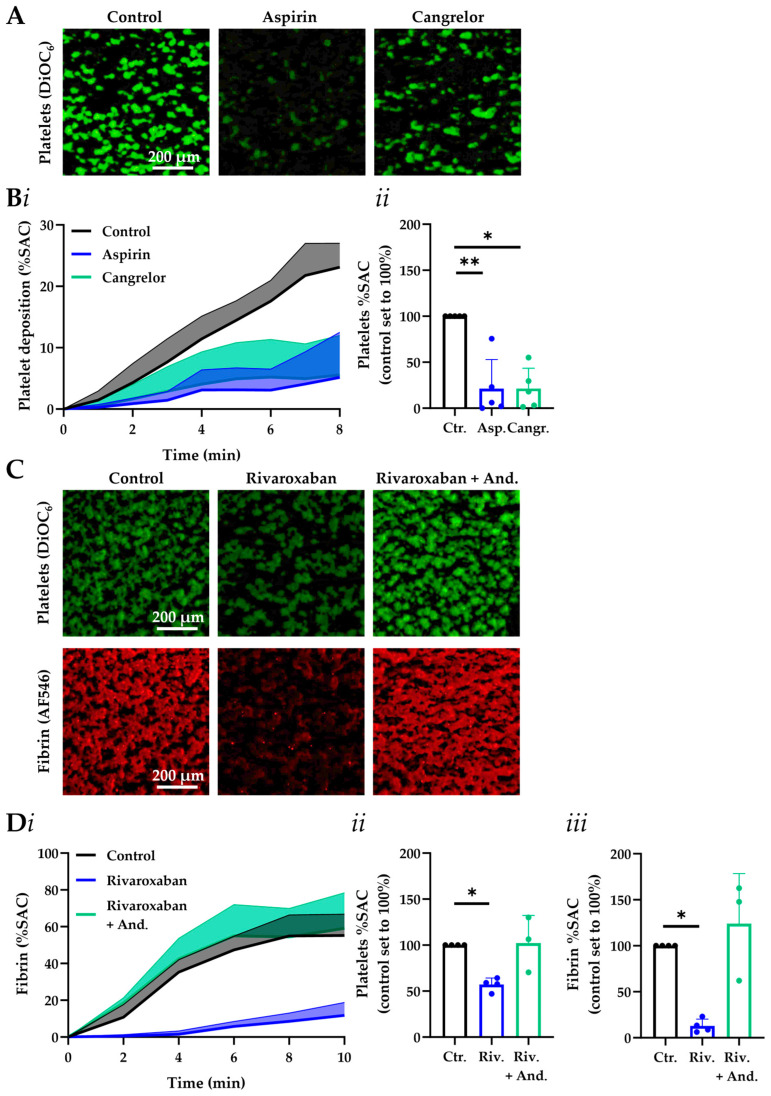
Sensitivity of MC-2S assessed by thrombus and fibrin formation at 37 °C for common anti-platelet and anticoagulant drugs. (**A**,**B**) Citrated blood samples were pretreated with aspirin (Asp., 100 μM) or cangrelor (Cangr., 0.5 μM) for 5 min. After labeling with DiOC_6_ (1 μg/mL), the blood was flowed through MS-2C chips coated with collagen-I for 8 min at wall-shear rate of 1000 s^−1^ (37 °C). Recalcification was not applied. Image capture was every 5 s. (**A**) Representative images of platelet thrombi formed after 8 min (bar = 200 μm). (**Bi**) Quantified traces of platelet deposition. Lower bold line indicates mean %SAC; upper line represents SD value. (**Bii**) Quantified inhibitor effects on platelet deposition after 8 min. (**C**,**D**) Citrated blood samples were preincubated for 5 min with rivaroxaban (Riv., 600 nM) and andexanet-α (And., 600 nM), as indicated. MC-2S chips were coated with collagen-I and tissue factor. After labeling with DiOC_6_ (1 μg/mL) and AF546-fibrinogen (15 μg/mL), blood was flowed under continuous recalcification for 10 min at 1000 s^−1^ (37 °C). Two-color fluorescence images were captured every 10 s. (**C**) The representative images of platelet thrombi and fibrin formed at selected endpoint (bar = 200 µm). (**D**) Time traces of fibrin formation (**i**). The lower bold line indicates mean %SAC and the upper line represents corresponding SD value. Effect of treatment on platelet deposition (**ii**) and fibrin formation (**iii**) at selected endpoint. The Kruskall-Wallis test with means + SD (five subjects) was used, and the significant values were indicated by * *p*-value < 0.05 and ** *p*-value < 0.01. For thrombus parameters, see Figures S2 and S3.

To evaluate the suitability of the system operating at 37 °C for platelet function analysis, we also compared the treatment of citrated blood samples with cangrelor (inhibiting P2Y_12_ receptors) in comparison to aspirin. Using the platelet membrane probe DiOC_6_, automated fluorescence image analysis showed a substantial suppression of thrombus formation in both treatment groups (Figure 3A,B), with reductions at 8 min for cangrelor of 79 ± 22% and for aspirin of 79 ± 32% (means ± SD, *n* = 5). Brightfield analysis of the thrombus morphological and contraction scores also indicated major inhibitory effects of cangrelor and aspirin (Figure S2A,B).

For comparison, collagen-coated MC-1 chambers were placed in a 37 °C environment, and similarly perfused with citrated blood at 1000 s^−1^. As expected, cangrelor and aspirin similarly suppressed thrombus formation with this conventional setup (Figure S2C). Collectively, these results demonstrate that the MC-2S chip operating at 37 °C reliably detected the antithrombotic effects of clinically relevant platelet inhibitors.

### 3.3. MC-2S Chip for Measurement of Coagulation Under Flow

The factor Xa inhibitor rivaroxaban is regularly prescribed for the prevention of recurrent myocardial infarction and atrial fibrillation [25,26]. In urgent cases, the antidote andexanet-α is given (a recombinant catalytically inactive form of factor Xa) [27], which antagonizes rivaroxaban and restores thrombin generation [28]. To evaluate the performance of the MC-2S chip in the setting of factor Xa inhibition, we examined the effects of both rivaroxaban and andexanet-α.

Thereto, blood samples were pretreated with a clinically relevant dose of rivaroxaban alone or in combination with andexanet-α at 1:1 molar ratio. Experiments using the MC-2S chip coated with collagen-tissue factor were performed at recalcified conditions and 37 °C. In the presence of rivaroxaban, platelet deposition under flow decreased with 43.0% (*p*-value < 0.01) and fibrin formation was essentially abolished (−87.2%, *p*-value < 0.001), when compared to the control condition (Figure 3C,D). The Supplementary Video S1 shows the accumulation of AF546-labeled fibrin on platelet thrombi, such as recorded by time-lapse imaging. Importantly, the extra addition of andexanet-α restored both platelet deposition and fibrin formation to control levels. The additional analysis of brightfield images revealed that scores for thrombus morphology and contraction rapidly increased for all conditions (Figure S3A,B). This suggests that the remaining thrombin activity, with inhibition of factor Xa activation by rivaroxaban treatment, suppressed the clotting process under flow, but still allowed (low) thrombin-dependent platelet deposition and aggregate contraction. This indicated effective detection of both anticoagulant and reverting procoagulant activity with the MC-2S chip. Parallel experiments using the MC-1 chamber, placed at 37 °C, yielded comparable results (Figure S3C).

### 3.4. MC-2S Chip for Measurement of Post-Thrombus Fibrinolysis

**Figure 4:**
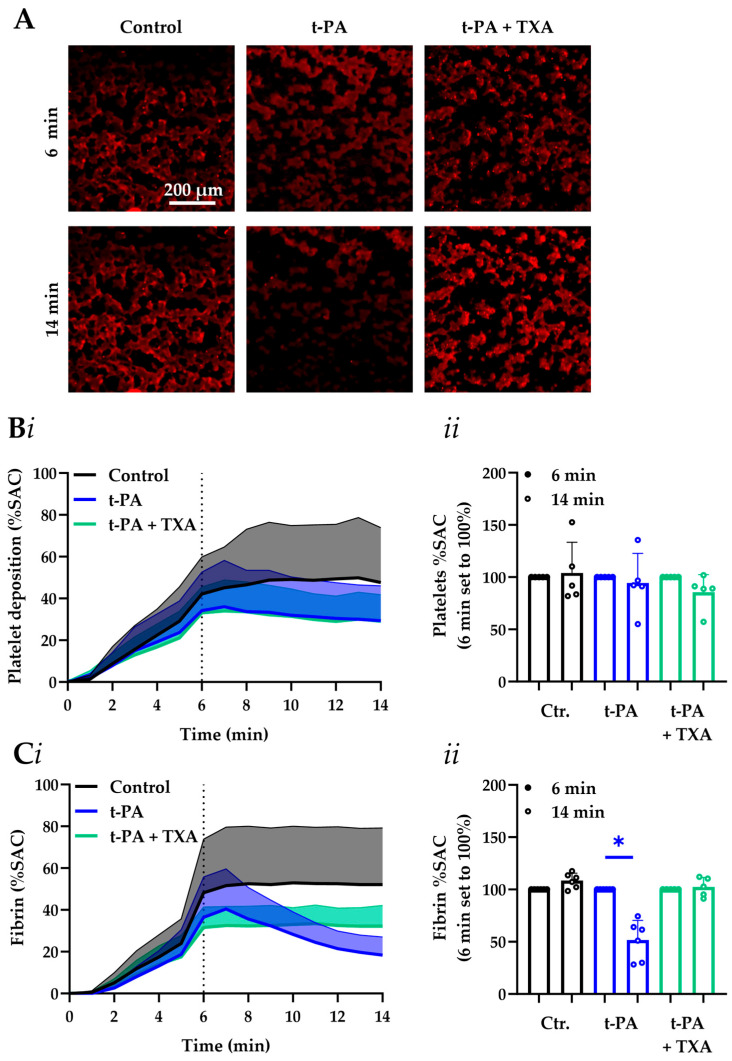
Sensitivity of MC-2S assessed by thrombus formation and fibrinolysis at 37 °C for commonly used drugs. (**A**–**C**) Citrated blood samples with low dose (3 nM) t-PA were pretreated with or without tranexamic acid (TXA, 50 μg/mL), as indicated. MC-2S chips were microspot-coated with collagen-I and tissue factor. After labeling with DiOC_6_ (1 μg/mL) and AF546-fibrinogen (15 μg/mL), blood was flowed under continuous recalcification for 6 min at 1000 s^−1^ (37 °C), after which perfusion for 8 min was continued using Hepes buffer with or without 75 nM t-PA. (**A**) The representative images of fibrin after 6 and 14 min (bar = 200 μm). Shown are time traces of platelet deposition (**Bi**) and fibrin formation (**Ci**). The lower bold line indicates mean %SAC and the upper line represents corresponding SD value. Effect of treatment on platelet deposition (**Bii**) and fibrin formation (**Cii**) at selected endpoint. The Wilcoxon test was used with means + SD (five subjects), and the significant values were indicated by * *p*-value < 0.05.

Tissue plasminogen activator (t-PA) is a potent inducer of the fibrinolytic pathway, whereas tranexamic acid serves as a clinically employed antifibrinolytic agent [21,29]. Using MC-2S chips, we quantified fibrinolysis under flow at 37 °C by first permitting the formation of platelet-fibrin thrombi for 6 min in the presence of a low dose of t-PA (3 nM), followed by replacement of the blood by buffer containing a high t-PA dose (75 nM) for an additional 8 min. We noticed that during the secondary perfusion phase, platelet deposition remained unchanged, whereas the massive fibrin content declined with 48% (*p*-value < 0.01) (Figure 4). Importantly, the t-PA effect was completely abolished by pretreatment of the blood with tranexamic acid (50 µg/mL). In parallel experiments using the MC-1 chamber, essentially similar results were obtained (Figure S4).

## 4. Discussion

The present findings demonstrate that the newly developed MC-2S microfluidic chip, connected to a small sized microscope, enabled rapid and sensitive assessment of platelet aggregation as well as secondary coagulation and fibrinolysis processes under relevant conditions of flow and temperature. By using defined thrombogenic surfaces (collagen with or without tissue factor), the platform provides a multidimensional readout of hemostatic regulation [30], dissecting the interplay between platelet activation, fibrin formation and clot dissolution. This triple approach contrasts with conventional hemostasis assays, which often conflate primary and secondary hemostasis and fail to capture the spatial and temporal complexity of thrombus formation [31,32,33]. An advancement is the operation at 37 °C, which contrasts with most existing flow chamber systems. Our data indicates that assaying at physiological temperature, to mimic in vivo conditions, enhances the sensitivity to clinically used antiplatelet drugs like aspirin and cangrelor. In line with earlier studies highlighting the temperature dependence of platelet signaling and thrombus architecture [19], our system uncovered functional differences that would otherwise remain masked. Additionally, coagulation dynamics appeared to be accelerated at 37 °C, while fibrinolytic responses remained unaffected.

The technical design of the MC-2S chips offers various translational benefits. The disposable and user-friendly construction simplifies assay execution, when compared to traditional chambers, and a relatively low blood volume is required. Moreover, the integration of the chip with lightweight imaging equipment and an automated image analysis script is supposed to facilitate adoption in research labs and for clinical workflows. Notably, the use for diagnostics requires establishment of reference ranges for given populations and working under standardized conditions—a need in current hemostasis testing [34].

Established platforms for hemostasis testing are the PFA-200, T-TAS and TEG (thromboelastography) systems. Our device has some specific differences and similarities in comparison to these platforms (Table 1). These are in particular the very high shear rates used in the PFA-200 system, limiting detection of antiplatelet drugs [35]. T-TAS, measuring flow-dependent thrombus formation dynamics in the thrombosis and hemostasis setting [5], does not provide insight into the thrombus architecture. Widely used in transfusion medicine [36], TEG assesses the viscoelastic properties of formed clots, yet in the absence of flow.

In this respect, the MC-2S system may serve as a versatile platform for functional diagnostics in patients with inherited bleeding disorders, platelet dysfunction, or unexplained coagulopathies. Its sensitivity to antiplatelet, anticoagulant, and antifibrinolytic agents also highlights a potential as drug-screening and monitoring tool, thereby complementing distinct assays such as thrombin generation tests and viscoelastic methods [36,37,38]. The system’s ability to model shear-dependent thrombus dynamics makes it particularly suited to studying thrombus formation at arterial flow conditions, where hemodynamic forces shape pathophysiology [39,40].

In conclusion, by uniting physiological flow, temperature control, and multidimensional hemostatic readouts in a disposable format, the MC-2S represents a next-generation microfluidic assay. This position is not only as a research tool for mechanistic dissection of thrombus biology but also as a candidate for point-of-care applications in personalized hemostasis and thrombosis risk evaluation. Future work should focus on validating the assay in clinical cohorts, establishing standardized cut-offs for pathological conditions, and benchmarking against existing diagnostic modalities.

## Figures and Tables

**Table 1:** Characteristics of MC-2S compared to established platforms for hemostasis testing.

	MC-2S	PFA-200	T-TAS	TEG
**Run** **time**	8 min	5 min	10 min	15–20 min
**Sample volume**	500 µL whole blood	800 µL whole blood	350 µL whole blood	2–3 mL whole blood
**Wall shear rate**	1000 s^−1^	5000–6000 s^−1^	2000 s^−1^	low
**Output type**	Kinetics of platelet and fibrin deposition (%SAC), thrombus morphology and contraction score	Closure time (CT) of platelet aggregates	Occlusion times (OC, OSC), thrombus profile (area-under-the flow-curve)	Clot elasticity initiation, clot propagation and lysis
**Application**	Haemostasis and thrombosis susceptibility	VWF and platelet activation test	Haemostasis and thrombosis susceptibility	Transfusion science and medicine

## Data Availability

The datasets used and/or analyzed during the current study are available from the corresponding author upon reasonable request.

## Supplementary Materials

The supporting information can be downloaded at: https://www.mdpi.com/article/10.3390/mi16111253/s1.
