# Supplementary material for: Automated Imaging and Analysis of Platelet, Coagulation and Fibrinolysis Activities Using a Novel Flow Chip-Based System at Physiological Temperature
Source: Micromachines (Basel). 2025 Oct 31;16(11):1253. doi: 10.3390/mi16111253 (PMC12654719; doi:10.3390/mi16111253)
Supplement: Supplementary file 1 [file micromachines-16-01253-s001.zip › Legends to supplementary figures and Table S1 .pdf]

Automated Imaging and Analysis of Platelet, Coagulation and Fibrinolysis Activities Using a Novel Flow Chip-Based System at Physiological Temperature

Xiang Gui [1,2], Bibian M. E. Tullemans [1], Bas de Laat [1], Johan W. M. Heemskerk [1] and Frauke Swieringa [1,*]

Legends to supplementary figures

Suppl. Figure S1. Design of MC-2S chip and predicted flow patterns. (A) Construction of y-shaped inlet tubing used for coagulation experiments (* connection to chip) (i) and longitudinal section through the MC-2S chip (ii) with blood connection port (a, 1 mm diameter), 15° angle of tubular to laminar chamber inlet (b), blood waste reservoir (c), and detection zone (d, field of view= 728 μm × 728 μm). (**B**) Modelled wall shear rates to achieve 1000 s^{-1} in MC-2S with chamber inlet of 15° (i) versus inlet of 90° (ii). For coagulation experiments, the y-shaped tubing (silicone Versitec, 1.0 mm internal diameter) had an inlet (0.5 mm opening) for injection of recalcification medium (1 volume, 0.45 mL/h) into the tubing of citrated blood (9 volumes, 4.05 mL/h). Further mixing was achieved by a 15° angle flow chamber inlet changing from a tubular (1 mm diameter) to a flat (50 μm height, 2 mm width) flow velocity profile.

Suppl. Figure S2. Suitability of MC-2S for assessment of platelet aggregation under flow under non-coagulant conditions at 37 °C. (A-B) Citrated blood samples were pretreated with aspirin (Asp. 100 μM) or cangrelor (Cangr., 0.5 μM) for 5 min, as for Figure 3 A-B. After labeling with $DiOC_6$ (1 μg/mL), the blood was flowed through MC-2S chips coated with collagen-I for 8 min at wall-shear rate of 1000 s^{-1} (37 °C). Recalcification was not applied. Brightfield and fluorescence images were captured automatically every 5 s. Shown are additional data to Figure 3 A-B, regarding analysis of thrombus morphology score **(A)** and thrombus contraction score **(B)** over time (i) and end values (ii). We refer to *De Witt, Nat. Commun. 2014, 5: 4257* for reference brightfield images of morphological and contraction scores. (**C**) Parallel blood perfusion experiments performed with conventional chamber MC-1, with equipment placed in 37 °C environment. Representative images of platelet thrombi formed (i, bar = 200 μm) and effect of treatment on platelet deposition at selected endpoint (ii). Mean ± SD (5 subjects), *P < 0.05, **P < 0.01, ***P < 0.001.

Suppl. Figure S3. Suitability of MC-2S for assessment of platelet-fibrin thrombus

formation under flow under coagulant conditions at 37 °C. (A-C) Citrated blood samples were preincubated for 5 min with rivaroxaban (Riv. 600 nM) and andexanet-α (And. 600 nM), as for Figure 3 C-D. MC-2S chips were coated with collagen-I and tissue factor. After labeling with $DiOC_6$ (1 µg/mL) and AF546-fibrinogen (15 µg/mL) blood was flowed under continuous recalcification for 10 min at 1000 s^{-1} (37 °C). Shown are additional outcome data to Figure 3 C-D, regarding morphological thrombus score **(A)** and thrombus contraction score **(B)** over time (i) and end values (ii). We refer to *De Witt, Nat. Commun. 2014, 5: 4257* for reference brightfield images of morphological and contraction scores. **(C)** Parallel blood perfusion experiments performed with conventional chamber MC-1, with equipment placed in 37 °C environment. Representative images of fibrin (i, bar = 200 µm) and effect of treatment on fibrin formed at selected endpoint (ii). Mean ± SD (5 subjects), ***P < 0.001.

Suppl. Figure S4. Comparative use of MC-1 for assessment of platelet-dependent fibrinolysis under flow at 37 °C. (A-B) Citrated blood samples were pretreated with 3 nM t-PA with or without tranexamic acid (TXA, 50 µg/mL), as indicated. MC-1 chips were coated with collagen-I and tissue factor. Samples supplemented with $DiOC_6$ (1 µg/mL) and AF546-fibrinogen (15 µg/mL) were flowed under continuous recalcification for 8 min at 1000 s^{-1} and 37 °C, after which perfusion for 8 min was continued using Hepes buffer with/out 75 nM t-PA. Shown are time traces of platelet deposition (A i) and fibrin formation (B i). Lower bold line indicates mean %SAC; upper line represents corresponding SD value. Effect of treatment on platelet deposition (A ii) and fibrin formation (B ii) at endpoint. Mean ± SD (5 subjects), *P < 0.05.

Suppl. Table S1: Experimental protocols for blood treatment with drugs.

Drug	Final concentration	Incubation time and temperature	Added to
Aspirin	100 µM	5 min, 37 °C	blood
Cangrelor	0.5 µM	5 min, 37 °C	blood
Rivaroxaban	600 nM	5 min, 37 °C	blood
Andexanet-α	600 nM	5 min, 37 °C	blood
t-PA	3 nM	5 min, 37 °C	blood
	75 nM	5 min, 37 °C	buffer
Tranexamic acid	50 µg/mL	5 min, 37 °C	blood

When two drugs were added to the same blood sample, a strict order of addition was maintained. In the coagulation mode, rivaroxaban was added before andexanet-α, whereas in the fibrinolysis mode, TXA was added before t-PA.
