# Supplementary figures and images for: Automated Imaging and Analysis of Platelet, Coagulation and Fibrinolysis Activities Using a Novel Flow Chip-Based System at Physiological Temperature
Source: Micromachines (Basel). 2025 Oct 31;16(11):1253. doi: 10.3390/mi16111253 (PMC12654719; doi:10.3390/mi16111253)

**Ai**

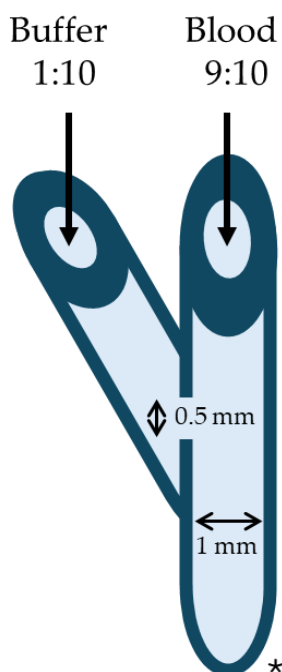

**ii**

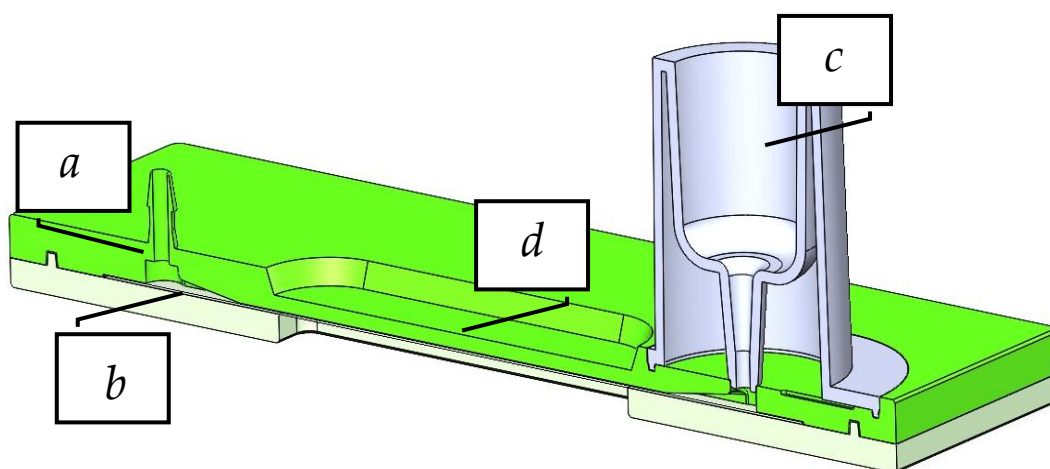

**Bi**

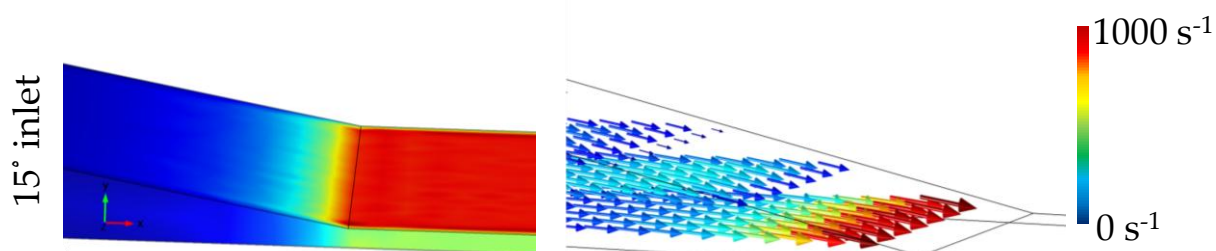

**ii**

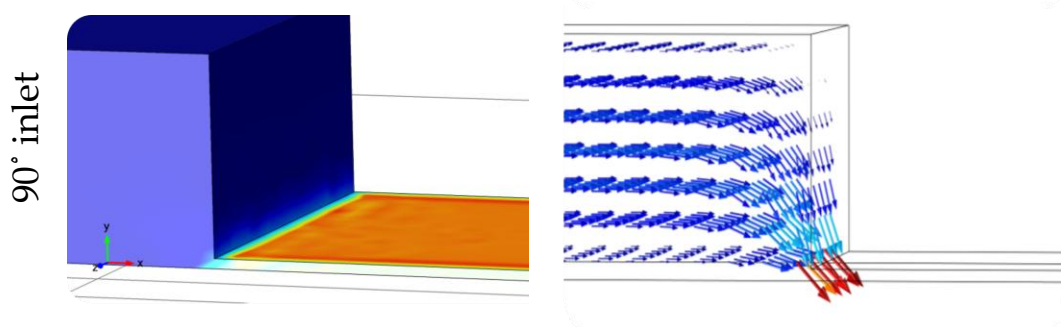

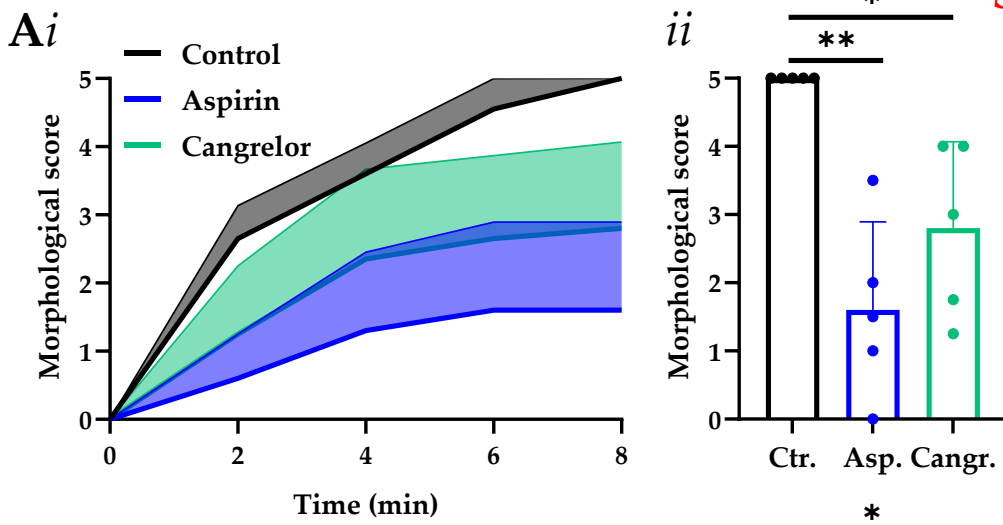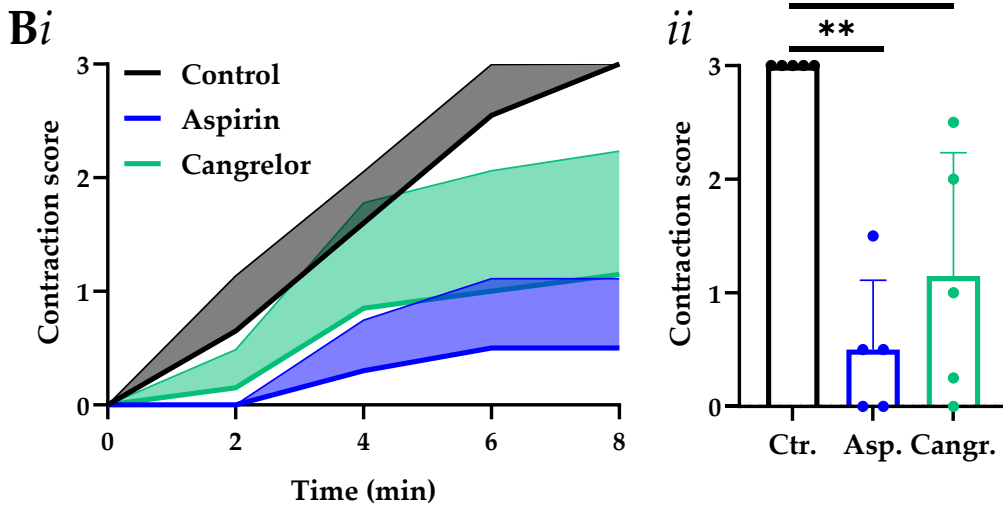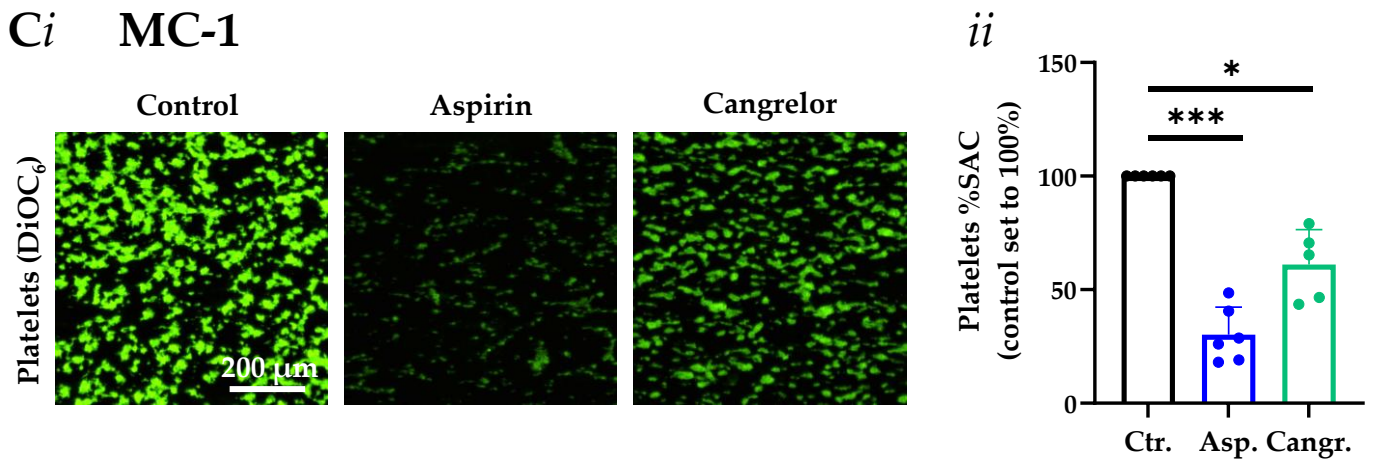

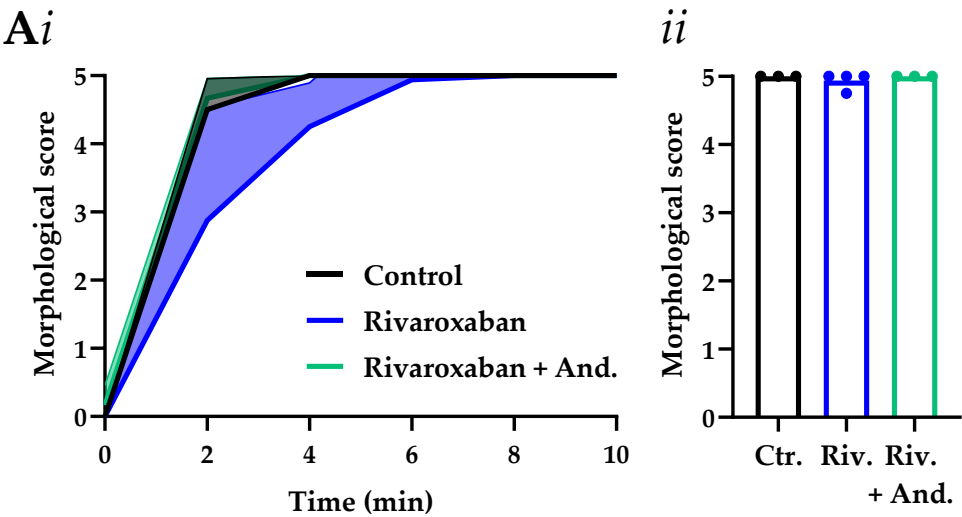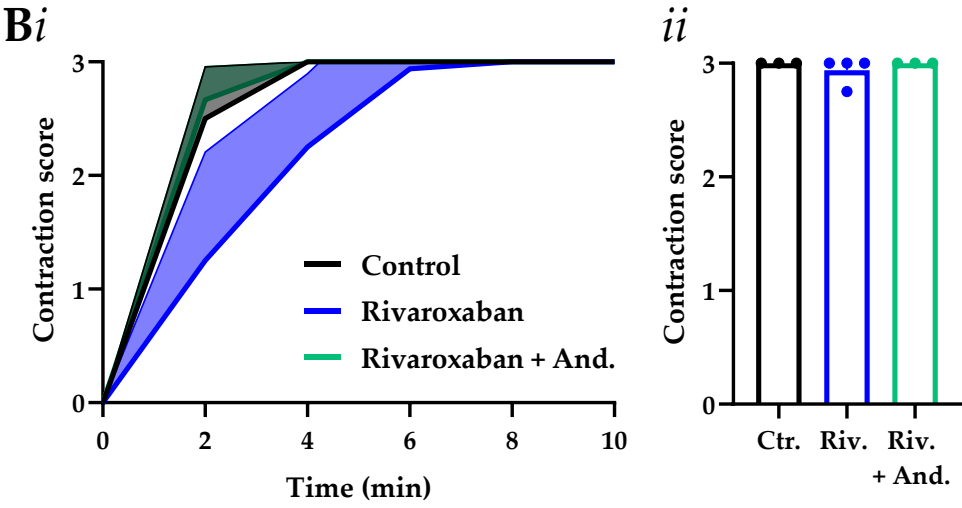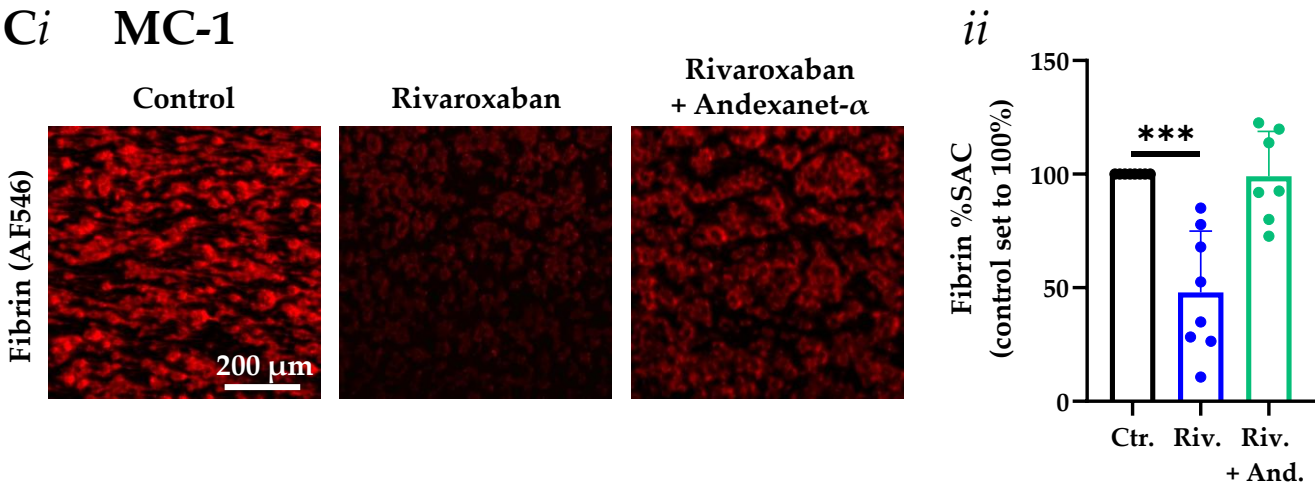

**Ai MC-1**

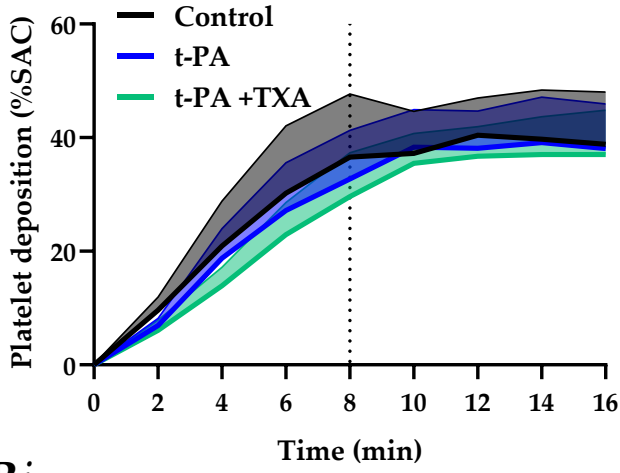

**ii**

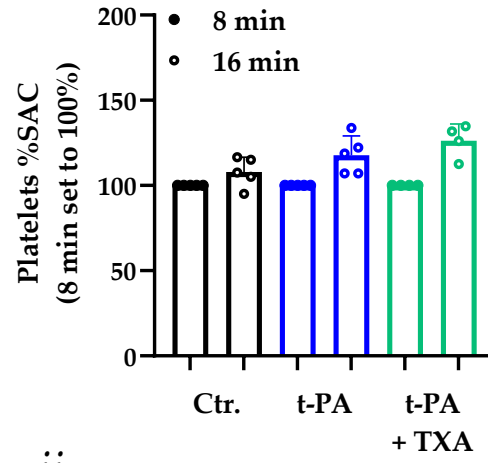

**Bi**

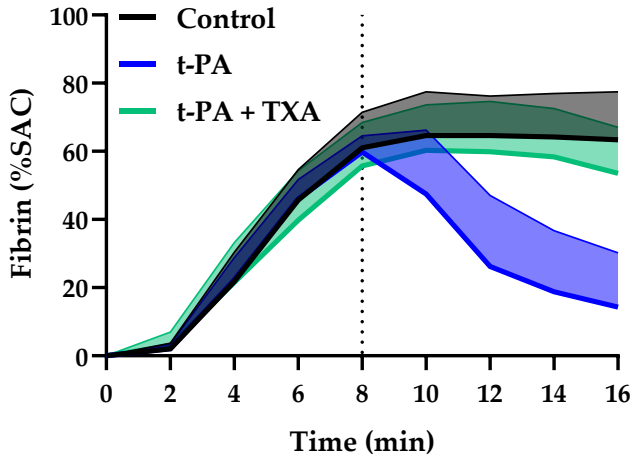

**ii**

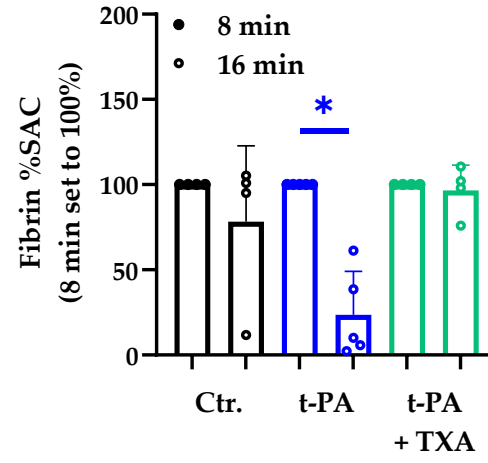

Supplement: Supplementary file 1 [file micromachines-16-01253-s001.zip › supplemental figures.pdf]
